# People’s perceptions on COVID-19 vaccination: an analysis of twitter discourse from four countries

**DOI:** 10.1038/s41598-023-41478-7

**Published:** 2023-08-31

**Authors:** Manah Verma, Nikhil Moudgil, Gaurav Goel, Peehu Pardeshi, Jacquleen Joseph, Neeraj Kumar, Kulbir Singh, Hari Singh, Prakash Babu Kodali

**Affiliations:** 1 Department of Computer Science and Engineering, Thapar Institute of Engineering and Technology, Patiala, Punjab 147004 India; 2 School of Energy and Environment, Thapar Institute of Engineering and Technology, Patiala, Punjab 147004 India; 3 Jamsetji Tata School of Disaster Studies, Tata Institute of Social Sciences, Deonar, Mumbai, 400088 India; 4 Tata Center for Technology and Design, Indian Institute of Technology Bombay, Mumbai, India; 5 School of Computer Science, University of Petroleum and Energy Studies, Dehradun, India; 6 Faculty of computing and IT, King Abdulaziz University, Jeddah, Saudi Arabia; 7 Department of Computer Science and Engineering, Graphics Era University, Dehradun, India; 8 Department of Electrical and Computer Engineering, Lebanese American University, Beirut, Lebanon; 9 Department of Civil Engineering, MM Engineering College, Maharishi Markandeshwar (Deemed to Be University), Mullana-Ambala, 133207 Haryana India; 10 Chemistry Department, RIMT UNIVERSITY, Mandi Gobindgarh, Punjab 147301 India; 11 Department of Public Health and Community Medicine, Central University of Kerala, Kasaragod, Kerala 671320 India

**Keywords:** Health care, Health policy, Health services, Public health

## Abstract

More than six and half million people have died as a result of the COVID-19 pandemic till Dec 2022. Vaccination is the most effective means to prevent mortality and infection attributed to COVID-19. Identifying public attitudes and perceptions on COVID-19 vaccination is essential to strengthening the vaccination programmes. This study aims to identify attitudes and perceptions of twitter users towards COVID-19 vaccinations in four different countries. A sentiment analysis of 663,377 tweets from October 2020 to September 2022 from four different countries (i.e., India, South Africa, UK, and Australia) was conducted. Text mining using roBERTA (Robustly Optimized Bert Pretraining approach) python library was used to identify the polarity of people’s attitude as "negative", "positive" or "neutral" based on tweets. A sample of 2000 tweets (500 from each country) were thematically analysed to explore the people’s perception concerning COVID-19 vaccines across the countries. The attitudes towards COVID-19 vaccines varied by countries. Negative attitudes were observed to be highest in India (58.48%), followed by United Kingdom (33.22%), Australia (31.42%) and South Africa (28.88%). Positive attitudes towards vaccines were highest in the United Kingdom (21.09%). The qualitative analysis yielded eight themes namely (i) vaccine shortages, (ii) vaccine side-effects, (iii) distrust on COVID-19 vaccines, (iv) voices for vaccine equity, (v) awareness about vaccines, (vi) myth busters, (vii) vaccines work and (viii) vaccines are safe. The twitter discourse reflected the evolving situation of COVID-19 pandemic and vaccination strategies, lacunae and positives in the respective countries studied.

## Introduction

Vaccination plays an important role in prevention of mortality and morbidity associated with infectious diseases. The ongoing Corona Virus Disease (COVID-19) had an unprecedented impact on global health systems and economies^1^. Achieving significant coverage of vaccination is the only long-term solution for the COVID-19 crisis. Internationally, the countries are focusing towards increasing the coverage of COVID-19 vaccination with booster dose being administered. COVID vaccines are now available for administration to individuals aged 5 years and above providing a significant protection from infection^2,3^. Studies indicate that Vaccines are effective in preventing COVID-19. However, despite vaccines being available in several countries, evidence of hesitancy towards COVID-19 vaccines emerge indicating it as a major public health challenge^4^.

Vaccine hesitancy is characterized as a delay in accepting or refusing vaccines despite the availability of vaccine services. It is impacted by factors such as complacency, indifference, and fear^5^. The World Health Organization (WHO) named “vaccine hesitancy” as one of the top global health threats^6^. Vaccine hesitancy and vaccine refusal is known to be associated with the outbreaks and reemergence of vaccine preventable diseases^4^. Intentional under vaccination was documented to be a reason for measles outbreaks even in highly vaccinated population^7^. Modelling studies reported that vaccine refusal can potentially lead to resurgence of infectious diseases such as measles, chickenpox and rubella with greater or severe illness^4^. Vaccine hesitancy is a key challenge in tackling the ongoing COVID-19 pandemic. Low vaccine coverage and vaccine hesitancy prolongs high incidence of COVID-19 cases, result in pocketed outbreaks and poses the risk of emergence of new strains of the infectious virus (e.g. BF.7). Evidence from European region reported the presence of mistrust and negative attitudes towards COVID-19 vaccines, even before the vaccines were publicly available^8^. Before the first vaccine was released, the percentage of individuals hesitant to take COVID-19 vaccines in Europe ranged between 13.9% and 43.7%^9–12^. In India, vaccine hesitancy rates among medical students were around 10.6%^13^. A 2020 global vaccine survey of 19 major economies reported that India and South Africa had vaccine acceptance rates of 74.53% and 79.79%, whereas UK had the acceptance rate of 71.48%^12^. In this regard, studying people’s attitudes towards vaccination is essential to inform public health decision making.

Social media is a major tool in new age public discourse including on key matters of public health importance such as vaccination. Studies have observed that the surge in public discourse on social media is linked to recent outbreaks of Ebola and Zika^14,15^. A recent systematic review concluded that individual networks within social media allow misinformation to flourish in the like-minded circles often resulting in pseudoscientific practices, conspiracy theories and hesitancy towards interventions such as vaccination^16^. Social media such as Twitter and Facebook served as key platforms for public discussion on the matters concerning COVID-19. A study analyzing over 45 million tweets during January 2020 to January 2021 reported a greater amount of twitter discourse on vaccine hesitancy and rejection compared to vaccine acceptance^17^. A recent study investigating 1.49 million unique tweets from 583,499 users from 11th March 2020 to 31st January 2021 reported that trust was the predominant emotion, followed by anticipation, fear and sadness^18^.

**Figure 1:**
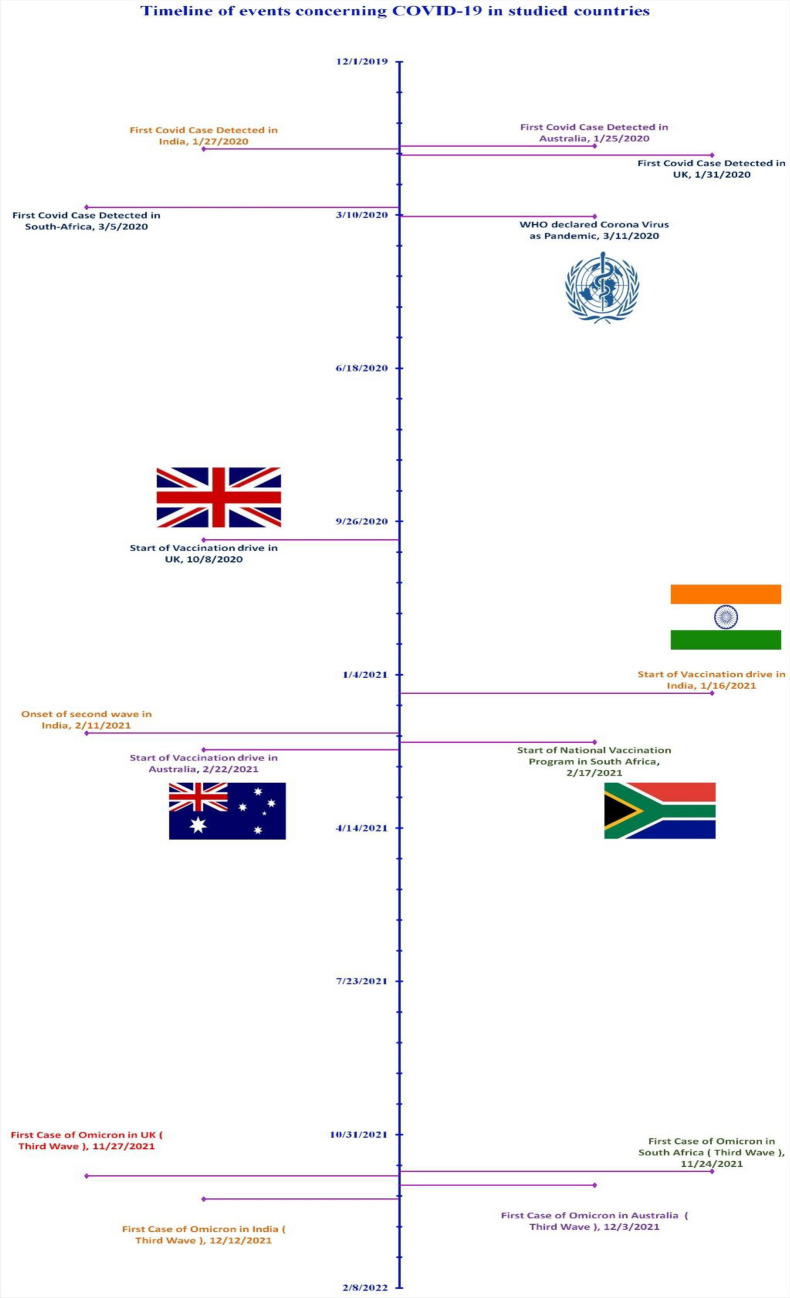
Figure outlining the timeline of events concerning COVID-19 in studied countries.

While the studies analyzing twitter discourse reported the varied underlying emotions, belief systems and attitudes towards COVID-19 vaccines, majority of the studies focused on twitter discourse up to January 2021. However, the accessibility and availability of COVID-19 vaccination globally improved only after March 2021. Moreover, several countries (including India & UK) witnessed multiple waves and variants of COVID-19 (Fig. 1). Understanding social media discourse in corroboration with developments in field of COVID-19 vaccination is helpful making conclusive interpretations of people’s perceptions towards COVID-19 vaccination programmes. Moreover, the observations can also facilitate improving the essential vaccination campaigns and predict the direction of public discourse in future outbreaks. The social media posts, specifically the twitter tweets are an excellent source of data to understand behavioral attitudes of a larger population concerning a specific idea.

Machine learning tools such as sentiment analysis are popularly used to analyse social media post and analyse people’s perception towards shared experiences such as vaccination. It is a systematic procedure of extracting, preparing and analyzing large volume of text data sharing similar characteristics (such as keywords and hashtags#). The countries India, South Africa, Australia, and United Kingdom are among the largest uses of twitter platform. The countries also account for among the highest doses of COVID-19 vaccines administered in their respective regions. Employing the tool of sentiment analysis, to analyse the tweets on COVID-19 vaccination in these countries the current study aims at addressing the following objectives.

1. To assess the attitudes towards COVID-19 vaccination in selected developed and developing countries.
2. To explore public perceptions on COVID-19 vaccination in the selected countries.

## Materials and methods

This research paper uses twitter data (i.e., tweets) to study the attitudes of people towards vaccination in different countries. Twitter is one of the most popular microblogging platforms, with a user base of 360 million active users in 2022. Countries were chosen so that analysis can be performed to study public perception towards COVID-19 vaccines in developed and developing country settings. India and South Africa were countries chosen to represent the developing countries whereas Australia and United Kingdom represented developed countries.

The data collected included the tweets extracted from the date of release of the first vaccine in the respective country till 30 September 2022. The dataset included all the tweets written in the English language related to the COVID-19 vaccine. The English language was preferred since the model used for sentimental analysis works only on tweets written in English. The tweets were analysed numerically and qualitatively.

### Numerical analysis

The numerical analysis of tweets was conducted to assess the attitudes towards COVID-19 vaccines across the countries. The numerical analysis followed the stages of (i) extraction, (ii) pre-processing and (iii) processing.

*(i) Extraction*

For extracting tweets, an automated web scraping tool known as “snscrape” [https://github.com/JustAnotherArchivist/snscrape] was used. Due to high pliancy, snscrape was the most appropriate and advanced scraping tool which directly searches the web for analyzing tweets of a certain region for a certain period. Snscrape has no limit on the number of tweets that can be extracted which makes it better than twitter’s own application programming interface.

Snscrape was configured according to the geographical location of the country, the date when the first vaccine was released there, and the language preferences. The raw data consisted of the union of all tweets, extracted using the query ‘COVID-19 Vaccine’ for each country.

*(ii) Pre-processing*

A tweet is a post on Twitter that can be up to 140 characters long, including spaces, emoji or Emoticon, hashtags, URLs, "@" mentions (Twitter handles), etc. Using REGEX (Regular expressions), these strings were removed from the tweets to make the data less chaotic before giving it as an input to machine learning algorithms. Regular expressions, also called REGEX, are used to search, extract, manipulate, and validate specific string patterns^19^. The tweets (which were not refined by snscrape) containing words from any other languages other than English were filtered out. After complete processing, a final number of 663,377 tweets were extracted for analysis. Country-wise distribution of the extracted tweets is shown in Table 1.

**Table 1:** Country-wise distribution of the frequency of all tweets for the selected countries.

S. No	Country	Date of Release of the first vaccine	Frequency of Tweets
1	India	16-01-2021	122,833
2	Australia	22-2-2021	226,510
3	South Africa	17-2-2021	125,581
4	United Kingdom	8-10-2020	188,453

*(iii) Processing*

We conducted sentiment analysis to identify attitudes of people regarding vaccination in developed and developing countries^20^. Based on the sentiments identified the attitudes of the twitter users based on the tweet was categorized into one of the three categories, i.e., positive, negative, or neutral. The RoBERTa (Robustly Optimized Bert Pre-training Approach) pre-trained model was used in this study. The pre-trained version of RoBERTa was trained on approximately 58 Million tweets^21^. Previously RoBERTa was found to have an 89% accuracy and 87% precision in undertaking sentiment analysis of COVID-19 tweets^22^.

The pre-processed tweets are passed to the tokenizer, which was a part of the pre-trained model. The encoded input is later passed into the RoBERTa model (pre-trained on tweets). The output was weights for the sentence to be negative, neutral, or positive, present at the[0][0] index of the output. The weights were extracted using detach function and converted into a numerical python array. Later softmax transformation was used to generate probabilistic overview for the country specific tweets being negative, neutral and positive. To ensure consistency and accuracy of the RoBERTa model in our study settings, we analysed a random sample of 400 tweets (100 per country) both manually and using RoBERTa and compared the findings. We computed Krippendorff’s alpha (α) to compare the agreement between manual estimation and RoBERTa. The value of α for positive tweets was 0.73, negative tweets was 0.74, and neutral tweets was 0.67 indicating a substantial agreement^23^.

Word cloud was generated from the tweets processed from each country to provide a visual representation of most frequent terms represented in tweets concerning COVID-19 vaccines.

### Qualitative analysis

The qualitative analysis was conducted to explore the views of public towards COVID-19 vaccination. The tweets were analysed manually to generate themes reflecting the public perception towards COVID-19 vaccines^24^.

A random sample of 500 tweets from each country (i.e., total of 2000 tweets) were extracted manually and analysed. Tweets which were in English, and having more than five words were included. Tweets which exclusively used non-alphabetical characters, religious/political campaigns and those with fewer words (less than five) were excluded. The extracted textual data in the form of tweets was inductively coded to develop initial codes. The codes were then processed to yield subthemes. The subthemes were amalgamated to form themes.

## Ethics declarations

### Ethical approval and informed consent

This study is a secondary analysis of a large dataset of over 0.66 million tweets between December 2020 and September 2022. Due to the vast volume of tweets and secondary nature of the data, obtaining informed consent from individual users was not practically feasible. As the data were publicly available tweets, there was no direct data collection from human participants. This eliminated the need for ethical clearance, typically required when collecting data directly from individuals. However, we ensured that all relevant ethical guidelines were adhered to while handling, analysing, and presenting the data.

First, all data extraction and curation processes were designed to exclude any personally identifiable information (PII) from the dataset. Specifically, the USER ID was removed from the dataset and replaced with a specific alphanumeric ID based on county and the serial number of extracted tweet. This was crucial to maintaining user anonymity and confidentiality. Moreover, during the analysis and presentation of the findings, great care was taken to present the results at a country level or in an aggregated form. This approach minimized the possibility of identifying any single user, further safeguarding the privacy of individuals contributing to the tweets in the dataset. Furthermore, the extracted data files were archived and stored securely after removing personal identifiers to maintain data security and confidentiality. Only the research team members had access to the data, and no information was shared with any third party outside of the research team. This strict control over data access and sharing contributed to the protection of user privacy. We ensured data minimization, extracting only the data directly relevant to the research questions, reducing the potential impact on user privacy. We respected user settings and adhered to the terms of service and privacy settings provided by the social media platform.

## Results

### Numerical findings

**Figure 2:**
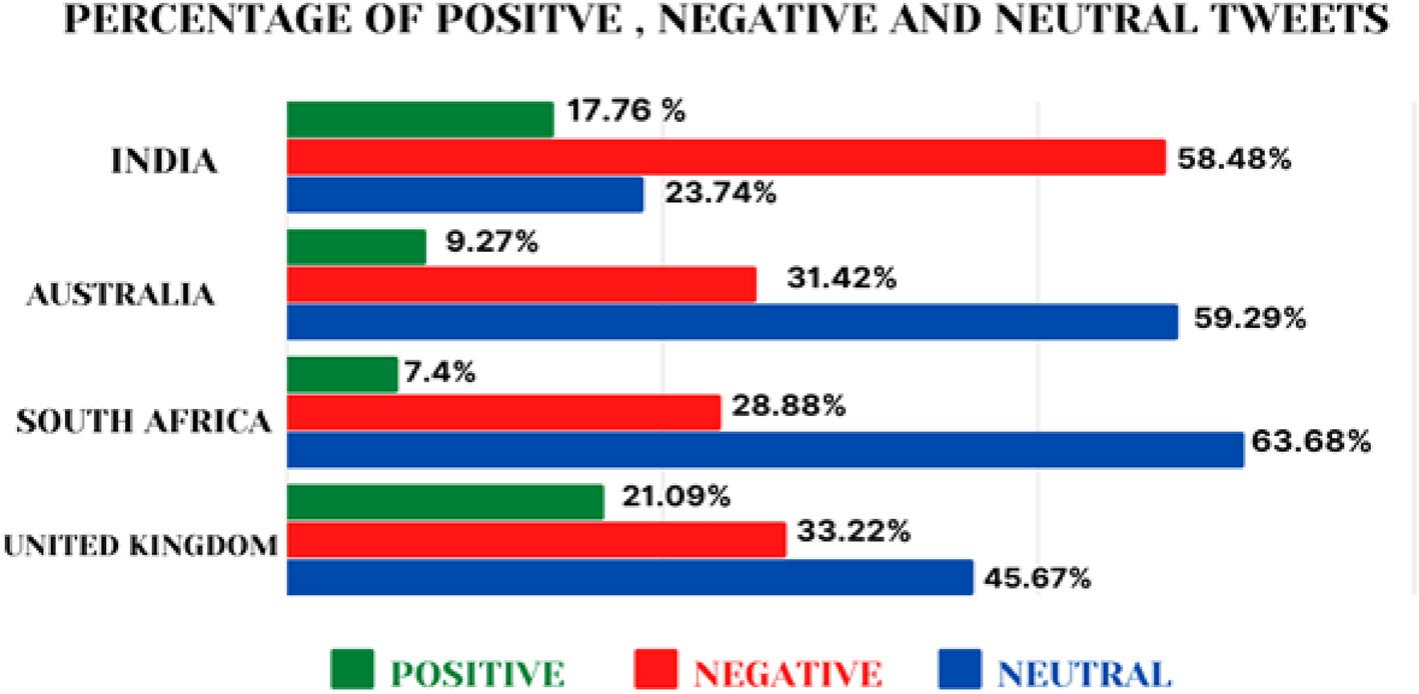
Graphical representation of percentage of negative, positive and neutral attitudes.

A total of 663,377 tweets were extracted and analysed. Figure 2 depicts the results of sentiment analysis of tweets from each country in terms of positive, negative as well as neutral tweets. While the maximum number of neutral tweets (have very little subjectivity linked to them) are from South Africa (63.88%), the minimum number of positive tweets (7.40%), as well as negative tweets (28.88%), were also extracted from South Africa only. India had highest percentage of negative tweets (58.48%) as compared to South Africa (28.88%). United Kingdom reported highest percentage of positive tweets (21.09%) as opposed to South Africa (7.40%).

**Figure 3:**
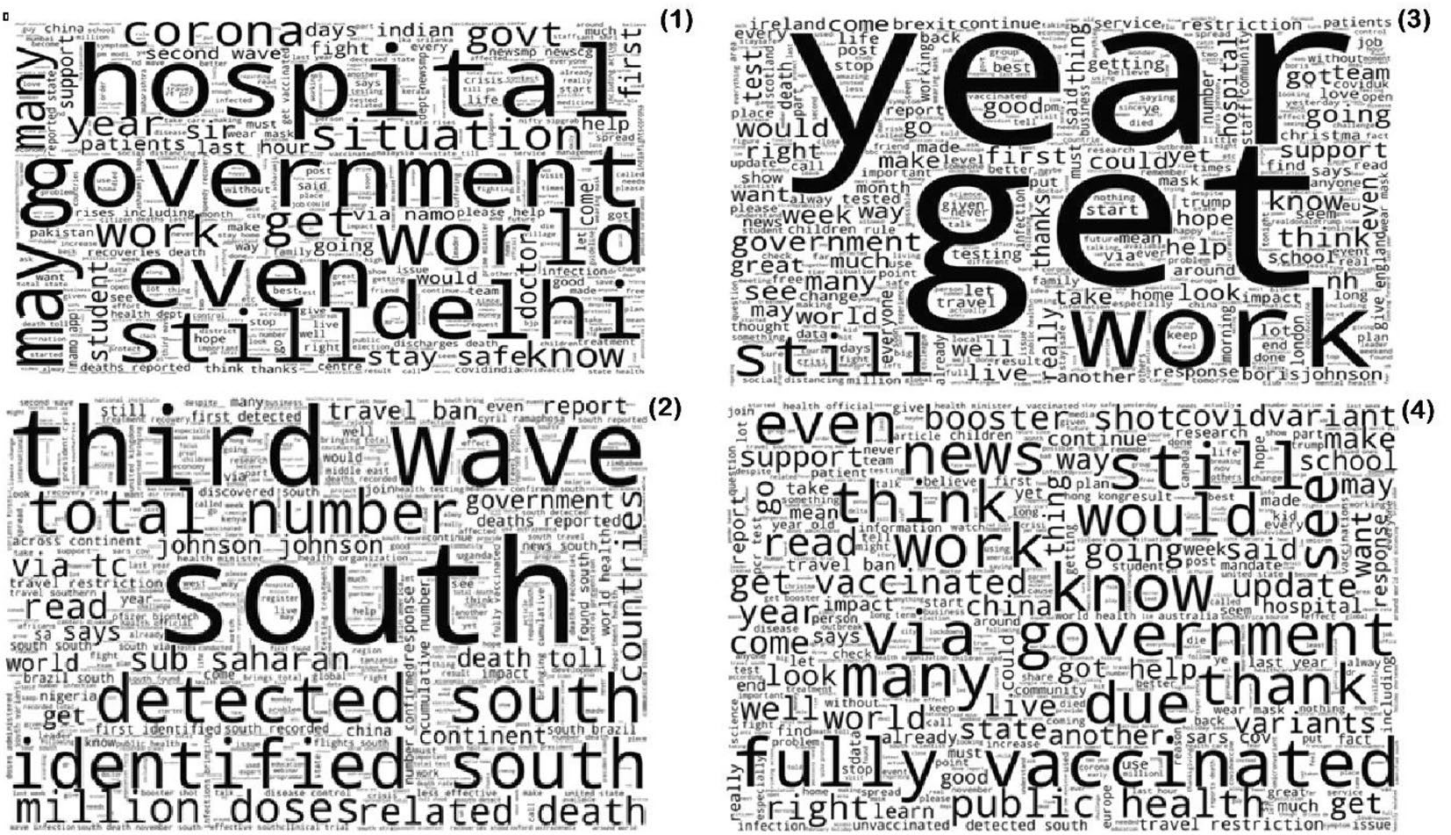
Word cloud generated by analysing the tweets. Footnotes: (1) India; (2) South Africa; (3) United Kingdom; (4) Australia.

The word cloud reflects the most frequently reported terms. In India, the words “government”, “Patients”, “Stay Safe”, “Second Wave”, “Hospital”, “Situation”, and “Help” were most frequently observed. In contrary, in Australia “Fully vaccinated”, “get vaccinated”, “variants”, “public health”, “support” etc. were most frequent words. Figure 3 outlines the word clouds from the four countries studied.

### Qualitative findings

**Figure 4:**
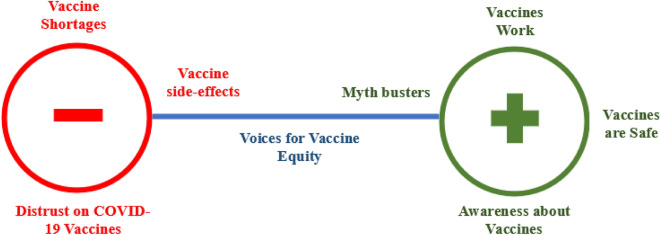
Figure outlining the themes identified across the polarity.

Several key themes emerged from the analysis of tweets sampled (Fig. 4). It was observed that the twitter served as a platform for advocacy, community engagement, information sharing, myth busting, awareness building, and reporting COVID-19 vaccination shortcomings. The themes reflected on the positive/negative attitudes of individuals towards COVID-19 Vaccines. Figure 4 outlines the themes emerged from the analysis of the tweets.

### Theme 1: awareness about vaccines

One of the commonly observed positive sentiment towards vaccination was spreading awareness about the COVID-19 vaccines, their availability and vaccination drives. Specifically, the twitter handles of national and local government machineries, civil society bodies and celebrities focused on sharing the information on vaccination, which were actively retweeted by other individuals. Such awareness about COVID-19 vaccines was observed alike for initial and booster doses of vaccines. One of the tweets from UK a country with a high vaccination rate said the following:

“Time to take up your #COVID19 booster jabs or your first or second vaccinations if you have not had them. If you have just become eligible for you #boosterjab wait to be contacted to invite you for vaccination. See below for current walk in vaccination sites. https://t.co/lLIkNlhlfx” (UK 05).

While countries like India faced vaccine shortages initially, the vaccination programmes amped as time progressed. Technology such as CoWIN platform was leveraged to aid the vaccination drive and develop awareness about vaccination.

“Book your vaccination on the given link, https://t.co/VkLfexCFiO, Current availability slot #Mumbai”(India 79583)

The information shared using the twitter handles served in better transmission & awareness about COVID-19 vaccination, positively contributing to vaccine coverage.

### Theme 2: vaccines work

A commonly observed positive tweets among twitter users is concerning the effectiveness of vaccines. The idiom “Vaccines Work” was a common catchphrase used by the national and regional governments, politicians, bureaucrats, health workers and celebrities across the countries. The users shared posters, web links, videos, statistics and research articles reporting efficacy and effectiveness of vaccines contributing to spreading a positive word about COVID-19 vaccines. Individuals even shared their real-life experiences touting that being unvaccinated increases the risk of COVID-19.

“I was shocked at the proportion of unvaccinated pregnant women now in ICU..….. Remember the vaccines work and are safer than encountering COVID19 first” (UK 7142).

“Half a million lives saved because of the COVID-19 vaccine. Vaccines work!” (Australia 108693).

As the COVID-19 pandemic progressed and new variants emerged the tweets were also observed to be reporting the effectiveness of vaccines on the multiple variants of COVID. Some of the tweets reporting effectiveness of vaccines towards new COVID-19 variants said the following.

“Good news! “Two doses of Pfizer jab are 90% effective against Covid-19 hospital admission for all variants for at least six months, according to a study”.”(UK 4670)

“Before anyone jumps to conclusion that @Pfizer-BioNTech #vaccine doesn't work against #COVID19 #DeltaVariant 3rd wave in #SouthAfrica. Data from #Israel show that it does. Spread the word. https://t.co/NbJ4XvJwNK” (UK 11971).

These messages enabled a positive discourse on the effectiveness of the COVID-19 vaccines, despite of the fears of new variants.

### Theme 3: vaccines are safe

The safety of the vaccines was another important domain found to be repetitive across the countries studied. Given the apprehensions surrounding the safety of COVID-19 vaccines, the twitter users largely advocated that the vaccines are safe to use. As the science of COVID-19 vaccines evolved the specific information concerning the safety of vaccines (among younger age groups, pregnant women etc.) was also reported. Twitter handles from India and South Africa tweeted the following.

“in South Africa, 71 k people have died of Covid-19, 7 million are Vaccinated and are fine a few with some side effects. It’s not rocket science, vaccinate.”(South Africa 42666).

“The fourth and the biggest phase of #COVID19 vaccination in India has commenced & according to #WHO, vaccines are safe to be administered to women on their periods. Don't delay your vaccinations.

Don't fall for myths.#VaccineForAll #VaccinationForAll https://t.co/r05KsgLpRg” (India 81029).

In India specifically, while initial concerns on vaccines such as “Covi-shield” were observed, the discourse gradually shifted to safety and efficacy of the vaccines. Twitter handles of popular leaders also facilitated in developing a positive discourse towards safety and efficacy of vaccines.

“There have been rumors about #vaccines made in India being unreliable. I would like to request all of you to stay aware and know that our vaccines are safe and effective: #PMModi”(India 71370).

Twitter served as a platform of advocacy for vaccine safety. Particularly the twitter handles of popular leaders and cultural icons vouching for vaccine safety improved trust and acceptance of vaccines in the community.

### Theme 4: voices for vaccine equity

The twitter users also advocated for vaccine sharing and vaccine equity to better control COVID-19 at a global scale. Specifically, the tweets were found to be advocating for vaccine equity by tagging the policy makers to share their stockpiles of vaccines with low-income countries through COVAX initiatives. One of the several tweets concerning vaccine equity tagging world leaders reads as follows.

“@BorisJohnson@JustinTrudeau@POTUS@EUCouncil@RegSprecher @ScottMorrisonMP the world needs #COVID19 vaccine access now. The #G7 must step up to #EndThePandemic for all by sharing 1 billion doses through #COVAX by September. https://t.co/ha2zi5IJEH” (UK 14594).

After emergence of new variants such as Omicron, the dialogue for vaccine equity was more strongly observed in the twitter discourse. The users voiced for vaccine sharing and vaccine equity for the developing countries to prevent emergence of newer variants of the virus.

One of the key lessons from the emergence of the new B.1.1.159 variant in Southern Africa is that if we don’t address the enormous global inequity in access to Covid-19 vaccines, other variants may emerge that are highly infectious or against which vaccines work less well. (Australia 89403).

### Theme 5: myth busters

A large proportion of positive tweets on vaccines were concerned with busting popular myths on COVID-19 vaccines. Specifically, in both developed and developing country settings, popular vaccine myths on conspiracy around COVID-19 vaccines, viability of COVID-19 vaccines for specific population groups, timing of vaccines and disease status etc. were debunked. Some of the popular tweets debunking COVID-19 vaccine myths are as follows.

“Covid-19 vaccines do not contain microchips https://t.co/kyWSAeqzDf via @harakahdailyHD” (India 980).

“MYTH: The vaccine alters your DNA.

FACT: Vaccines do not change a person’s DNA. Vaccines work by stimulating the body the same way the virus would if someone was infected.”(Australia 98087).

Debunking COVID-19 vaccine myths was also actively done by the Government agencies across the countries. For example, the Government of India as part of its COVID-19 prevention campaign, launched “Jan Andolan” to bust myths and spread awareness about COVID-19 vaccines.

“Mythbusters on #COVID19Vaccines.

Myth: Vaccine not needed for #COVID19 recovered persons.

Fact: Advisable to receive a complete schedule of vaccine despite recovery from COVID-19, to enhance the immune response. #JanAndolan @SpokespersonMoD @diprjk @PIB_India @rajnathsingh https://t.co/hkWDXoJOFZ” (India 120860).

The active participation of individuals, government and civil society bodies, prevented fear & panic and contributed to the positive discourse on COVID-19 vaccines.

### Theme 6: vaccine shortages

Experiences of shortage of vaccines were among the common negative discourse observed in developing countries. In Indian context, the tweets on vaccine shortages raised exponentially during the April-June 2021 quarter. One tweet concerning suspension of vaccination drive read as follows.

“COVID-19 vaccination drive in Mumbai suspended for two days due to vaccine shortage https://t.co/K6eSNlssJx” (India 52738).

In developed countries like UK while the vaccine shortages are substantially less visible, the tweets reflected the user’s advocacy to tackle vaccine shortages in developing countries, specifically in Africa.

“Everyone vaccinated on an remote island in Scotland that had no #COVID19 cases… instead of giving the vaccine to individuals in #COVID19 UK hotspots or sharing vaccine with other countries? There is a global vaccine shortage! This is #vaccinenationalism at its finest! https://t.co/HxZyhvOf09” (UK 19740).

The tweets concerning vaccine shortages also reflected on the supply chain & health system issues within the countries and larger vaccine inequity between the developed and developing countries. One twitter handle from South Africa said.

“WHO warns lack of COVID-19 vaccine supply in Africa could make it breeding ground for new variants and send the whole world back to square one” (South Africa 29430).

### Theme 7: distrust on vaccines

The negative discourse on COVID-19 vaccines also reflected the general distrust of COVID-19 vaccines in the communities. The distrust of vaccines is largely reported in the developed country settings, quoting concerns around expedited process of vaccine development, capitalism and big pharma lobbying. The twitter discourse reflected the user’s skepticism on COVID-19 vaccines and their development process. Some tweets from Australia and the UK read as follows.

“So it seems people are finally realizing the covid 19 vaccines aren’t vaccines. They just call these shots vaccine so people who get serious side effects can’t sue the gov. Covid19 is just a shot people.#shots#vaccines#thanksgivingconvos https://t.co/lRhqrOW9gu” (Australia 46606).

“People are not linking a drug and vaccine. They're linking what can happen if pharmaceutical companies don't trial and test a product adequately. AIDS has been around for 50 years but no vaccine whereas COVID-19 about a year. Public have a right to be concerned” (UK 86542).

“There are no vaccines available for most of NTDs, Malaria, HIV and others, but there are many vaccines developed and manufactured in less than one year time against #COVID19. #RuleOf3Ps Fact speaks itself” https://t.co/01C0kFaQju” (UK 20113).

In developing countries, the vaccine distrust was largely observed in villages and areas with limited awareness. In rural India, distrust was mainly surrounded by the myths that COVID-19 vaccines will cause infertility and impotency.

“We are hearing the rural population is scared of the vaccine—they think it will make women infertile and men impotent. Children are being sent away when there is a vaccine campaign in villages.#COVID19 #VaccineHesitancy #mythbusting” (India 79298).

### Theme 8: vaccine side-effects

The distrust on vaccines was seen to be largely linked to the fear of vaccine side-effects. In this regard it was observed that twitter also served as platform to share reliable information and updates on rare events such as vaccine side-effects. The platform served to communicate any recommendation, report, news concerning vaccine side-effects such as allergic reactions, bell’s palsy, and even death in the countries studied. Some of the tweets studied are as follows.

“2 people have experienced allergic reactions and 4 people have developed Bells' palsy to Pfizer's COVID19 Vaccine so far”. (UK 70882).

“Covid Has A 99% Survival Rate.

Now I personally know of at least two otherwise healthy people who died of blood clots after getting the vaccine & three others who developed Bells Palsy.

https://t.co/40z6yv39n3″ (Australia 116251).

“#COVID19 | Government’s review of side effects confirms 1 death after vaccine dose” (India 68330).

## Discussion

The study analysed the twitter discourse & people’s sentiments on COVID-19 vaccines in four selected countries. Across the counties there was a general negative sentiment on COVID-19 vaccines ranging between 28.88 and 58.48%. The highest percentage of tweets with negative sentiments were identified from India, whereas the highest percentage of positive tweets were identified in UK i.e., 21.09%.

The tweets reflected the general reality of vaccination status and the overall discourse around the vaccination in specific country during the time period. For example, the tweets in India reflected the words “Second wave”, “Delhi”, and “Stay safe” a reminiscence of the deadly second wave the country witnessed in mid-2021^25^. In UK the prominent words “Get”, “Work” reflects the government’s advocacy that “vaccines work” and “get vaccinated” the prominent messages communicated by the UK government during that time. In South Africa, the prominent words were “detected south”, “identified south”, “million doses” and “Johnson & Johnson” conveying the messages concerning the challenges in vaccine roll out in the country^26^.

**Figure 5:**
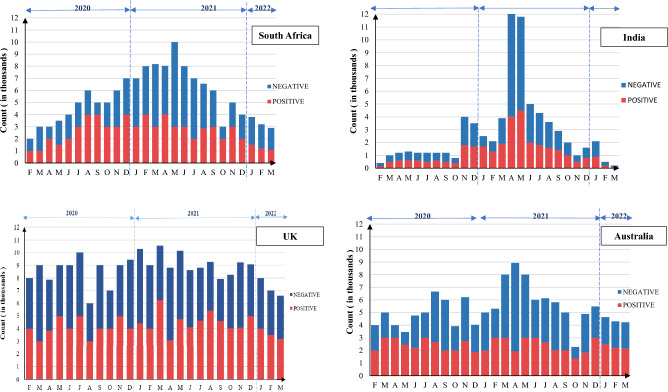
Positive and negative tweets across COVID-19 timeline in selected countries.

From the overall discourse we observed that the major negative themes emerged included (i) vaccine shortages, (ii) distrust on vaccines and (iii) side effects. In developing countries, the discourse was largely concerning the supply side factors of vaccines i.e., shortage of vaccines. The attitudes observed in twitter discourse were confirmatory with the COVID-19 status and vaccine rollout in respective countries. The data on weekly confirmed COVID-19 mortality indicates that India witnessed the highest number of deaths during the study timeline (supplementary Fig. 1A), explaining the high percentage of negative tweets. The COVID-19 vaccine doses remained administered per 100 people in South Africa remained less than 65/100 during the study period (supplementary Fig. 1B), indicating substantial challenges in vaccine delivery. Our findings suggest that high number of COVID-19 deaths, coupled with systematic inefficiencies resulted in negative attitudes. This is further supported by our supplementary analysis reflecting the tweets across COVID-19 timeline in India (Fig. 5). We found that the negative tweets peaked during the months of April to June 2021, during which India witnessed a massive spike in deaths due to COVID-19. The content of these tweets was “medicine stock outs”, “vaccine shortages”, “requests for help”, “shortage of hospital beds”, and “systematic failure”. The word cloud for India (Fig. 2(1)) supports the same.

Our findings reflect that twitter was also used as a platform to voice for vaccine equity. Users from developed countries were observed to call out the leaders to share their surplus vaccines with developing world through COVAX initiative. Studies argue that health system inadequacies, coupled with limited vaccine supplies, high percentage of susceptible population, and increase in virulent variants of virus can result in potential outbreaks of COVID-19 in the developing countries^27^. While Vaccination, is the most effective strategy to prevent infection, the unavailability of COVID-19 vaccines still remains a major problem three years into the pandemic. While 68.7% of the world population received at least one dose of COVID-19 vaccination, less than 26% of those in low-income countries received it^28^. Vaccination shortages and logistic inefficiencies are reported as reasons for inadequate vaccination rates in low- and middle-income countries of Asia and Africa^29,30^. Our findings reflect that from a developing country perspective, availability and accessibility of vaccines should be of prime focus.

In Australia and the United Kingdome which had a high vaccination rates (supplementary Fig. 1B), the discourse was largely concerning the trust-worthiness and side effects of vaccines. Specifically, the swiftness at which the COVID-19 vaccines were developed, coupled with the myths surrounding them created a sense of distrust. Existing research from UK and elsewhere corroborate with our study findings. A study conducted in France reported that 28.8% of working age adults out rightly refused the COVID-19 vaccination^31^. Another study reported that 40.9% of the US population had a low level of trust on COVID-19 vaccine^32^. In India among the population groups such as medical community who were priority recipients of COVID-19 vaccines during India’s initial COVID-19 vaccination drive, vaccine hesitancy was reported to be around 10.6%^13^. A recent study on vaccine hesitancy among UK adults reported that existing misinformation, concerns about vaccine safety and personal beliefs impacted vaccination decision making^33^.

To improve uptake of COVID-19 vaccines, countries have adopted multiple strategies to spread awareness about safety of vaccines, and busting the myths around vaccination. Individual and governmental effects were observed towards communicating a positive word on COVID-19 vaccines through platforms like twitter. In developed and developing countries alike, there were several myths such as “vaccines having microchips”, “those with prior COVID-19 infection need not take vaccine”, “COVID-19 vaccine causes infertility” etc., which hindered vaccine acceptance at regional and national levels^33,34^. Twitter served as one of the platforms to bust the myths. Specifically, the official handles of government agencies were found to bust specific myths surrounding COVID-19 vaccines across the countries. Earlier studies also report the use of social media to counter misinformation and disseminate accurate information^35–37^.

Similarly, twitter served as a platform to disseminate information on vaccine safety by popular leaders and celebrities. Popular leaders such as the Indian and UK prime ministers posted on twitter taking their first doses of COVID-19 vaccines creating a positive image for vaccines. Earlier studies reported that politicians and celebrities personally taking the vaccine and urging the fellow countrymen to take the vaccine created a positive image and confidence on COVID-19 vaccines^38,39^. Additionally, it can be argued that twitter served as a means to undertake persuasive messaging, by spreading the positive messages such as “vaccines work”, “get the jab done”, “vaccines are safe” etc. Studies report that persuasive messaging is effective in increasing the vaccination uptake and improve awareness^40^.

## Limitations

This study focused solely on four countries and may not provide a generalizable view to global discourse on COVID-19 vaccines. Twitter was the primary data source for the study. Non-inclusion of other social media platforms, restrictions on length of tweets, and confirming to tweets in English language are some of the limitations with the data. The study could not establish causal associations given its use of a single data source and descriptive focus of objectives and analysis. Moreover, social media data such as twitter possess the risk of sampling bias, language and cultural biases, geographical bias, and echo chamber effect which might limit representativeness and generalisability. We attempted to overcome these limitations by analysing a large sample more than 100,000 tweets per country, purposively identifying the major economies from Europe, Oceania, South Asia, and Sub-Saharan Africa, and stratifying the results of our sentiment analysis by geography. Albeit a smaller sample, by thematically analysing 500 randomly selected tweets from each country, and contextualising our analysis with the real word COVID-19 data we complemented our numerical analysis and minimized the potential bias of echo chamber effect. Future studies triangulating the sentiment analysis with multi-site qualitative interviews or thematic analysis of a larger sample of tweets could provide further insights. Studies employing longitudinal approaches are needed to establish temporality and causation. In spite of limitations, this study provides a crucial insight to public opinion on COVID-19 vaccines in the countries studied.

## Conclusion

Our findings suggest that twitter discourse reflected the general trend in progression of COVID-19 epidemic and vaccinations in the studied countries. While, there exist a substantial negative perception among the population, the negative sentiments were usually backed by vaccine shortages and supply chain inefficiencies in developing countries and safety concerns and myths surrounding vaccines in developed countries. These findings reflect the core issues which are faced in the specific countries studied. In developed countries with stronger healthcare systems and better availability of vaccines, the focus should be to improve public perception on vaccination. In contrary, more efforts are needed to strengthen the vaccine availability, delivery systems, and improving public perception in developing countries. The findings also suggest the role of social media as platforms to share a positive word about vaccination and suggest a future potential role of social media in tackling misinformation. Given that the threat of COVID-19 is still looming and inadequate vaccination is a persistent challenge, social media should be continued to be used by concerned authorities to report positive messages on COVID-19. Also, the findings point towards the prominence of analysing the discourse in social media to inform public health strategies towards disease prevention and management.

### Supplementary Information

Supplementary Information.

## Data Availability

The datasets used and/or analysed during the current study available from the corresponding author on reasonable request.
