# Supplementary material for: People’s perceptions on COVID-19 vaccination: an analysis of twitter discourse from four countries
Source: Sci Rep. 2023 Aug 31;13:14281. doi: 10.1038/s41598-023-41478-7 (PMC10471683; doi:10.1038/s41598-023-41478-7)
Supplement: Supplementary file 1 — Supplementary Information. [file 41598_2023_41478_MOESM1_ESM.pdf]

Title: People's perceptions on COVID-19 vaccination: An Analysis of twitter discourse from four countries

Supplementary File: Trends in COVID-19 deaths and Vaccine doses in the countries studied.

A

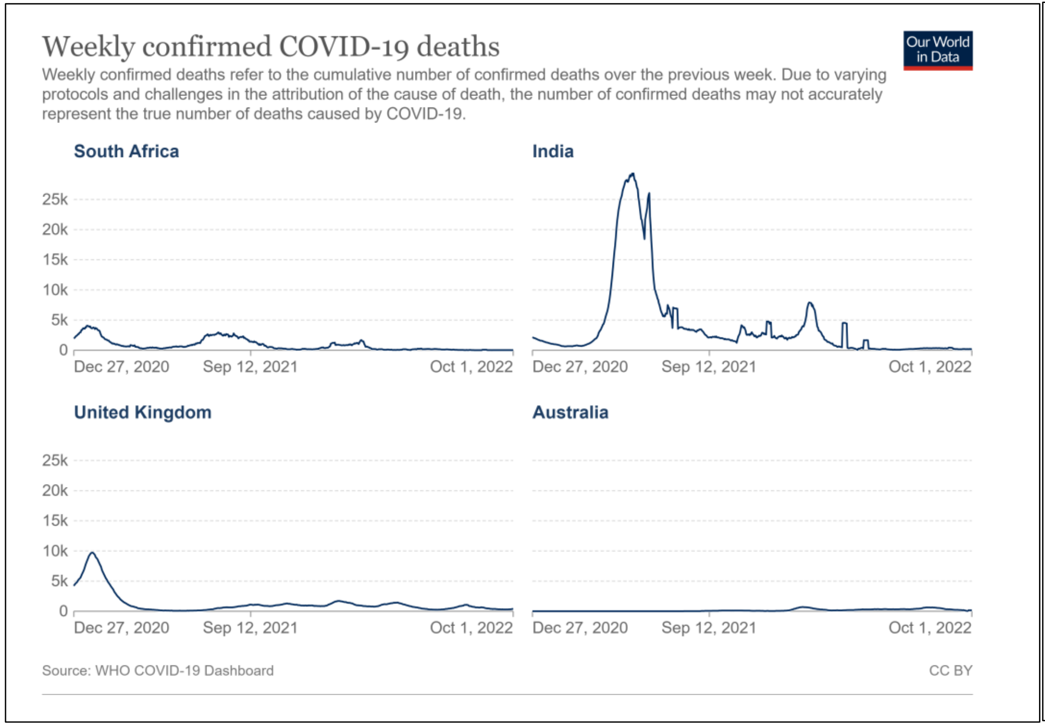

B

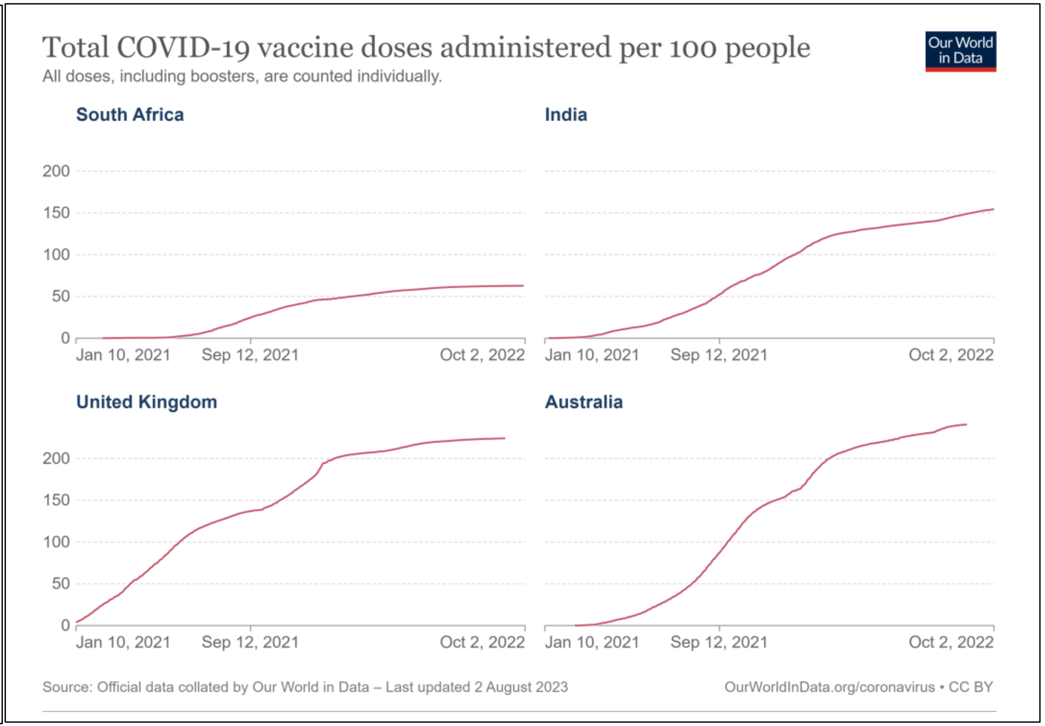

Source: Edouard Mathieu, Hannah Ritchie, Lucas Rodés-Guirao, Cameron Appel, Charlie Giattino, Joe Hasell, Bobbie Macdonald, Saloni Dattani, Diana Beltekian, Esteban Ortiz-Ospina and Max Roser (2020) - "Coronavirus Pandemic (COVID-19)". Published online at OurWorldInData.org. Retrieved from: 'https://ourworldindata.org/coronavirus' [Online Resource]
